# Highly active CRISPR-adaptation proteins revealed by a robust enrichment technology

**DOI:** 10.1093/nar/gkad510

**Published:** 2023-06-16

**Authors:** Ido Yosef, Tridib Mahata, Moran G Goren, Or J Degany, Adam Ben-Shem, Udi Qimron

**Affiliations:** Department of Clinical Microbiology and Immunology, Sackler School of Medicine, Tel Aviv University, Tel Aviv 69978, Israel; Department of Clinical Microbiology and Immunology, Sackler School of Medicine, Tel Aviv University, Tel Aviv 69978, Israel; Department of Clinical Microbiology and Immunology, Sackler School of Medicine, Tel Aviv University, Tel Aviv 69978, Israel; Department of Clinical Microbiology and Immunology, Sackler School of Medicine, Tel Aviv University, Tel Aviv 69978, Israel; Department of Integrated Structural Biology, Equipe labellisée Ligue Contre le Cancer, Institut de Génétique et de Biologie Moléculaire et Cellulaire, Illkirch 67404, France; Department of Clinical Microbiology and Immunology, Sackler School of Medicine, Tel Aviv University, Tel Aviv 69978, Israel

## Abstract

Natural prokaryotic defense via the CRISPR–Cas system requires spacer integration into the CRISPR array in a process called adaptation. To search for adaptation proteins with enhanced capabilities, we established a robust perpetual DNA packaging and transfer (PeDPaT) system that uses a strain of T7 phage to package plasmids and transfer them without killing the host, and then uses a different strain of T7 phage to repeat the cycle. We used PeDPaT to identify better adaptation proteins—Cas1 and Cas2—by enriching mutants that provide higher adaptation efficiency. We identified two mutant Cas1 proteins that show up to 10-fold enhanced adaptation *in vivo*. *In vitro*, one mutant has higher integration and DNA binding activities, and another has a higher disintegration activity compared to the wild-type Cas1. Lastly, we showed that their specificity for selecting a protospacer adjacent motif is decreased. The PeDPaT technology may be used for many robust screens requiring efficient and effortless DNA transduction.

## Graphical Abstract

**Graphical Abstract:**
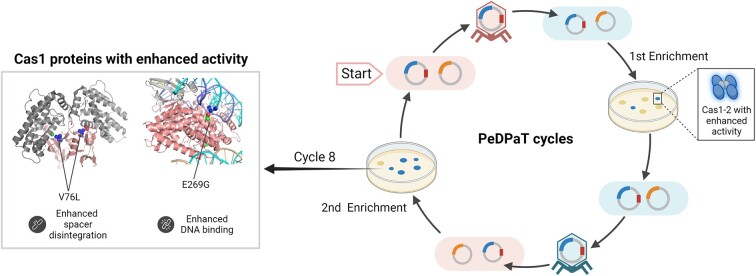

## INTRODUCTION

Components of clustered regularly interspaced short palindromic repeats (CRISPR) and their associated proteins (Cas) have been identified as central to prokaryotic immune systems (1). Various types of CRISPR–Cas systems (2,3) defend prokaryotes against horizontally transferred DNA (1,4,5) and RNA (6–8). The immunity relies on small CRISPR RNAs (crRNAs) that guide Cas protein(s) to cleave complementary nucleic acids in a sequence-specific manner (1,4–6,9). The genetic loci of all systems include a CRISPR array—short repeated sequences, called ‘repeats’, that flank similarly sized sequences, called ‘spacers’. The spacers are acquired from DNA sequences termed ‘protospacers’. Their incorporation into the bacterial CRISPR array, termed ‘adaptation’, creates a repertoire that guides the system against invaders in subsequent encounters. To identify a suitable protospacer for adaptation, many CRISPR systems employ short 3- to 7-bp protospacer adjacent motifs (PAMs) (9–14). These motifs differ from those present in the repeats; their absence in the repeats thus prevents an attack on the bacterial CRISPR array. Following protospacer selection and processing, the adaptation machinery performs site-specific integration of the new spacer into the CRISPR array. Protection from target DNA is conferred after the crRNA is processed by Cas proteins into RNA-based spacers flanked by partial repeats. These RNA spacers specifically direct Cas interference proteins to target and cleave nucleic acids encoding matching protospacers. Thus, the system can adaptively and specifically target invaders.

The adaptation process has been thoroughly characterized for many different CRISPR–Cas systems (15–18). In the *Escherichia coli* type I-E CRISPR–Cas system, two proteins, Cas1 and Cas2, are both necessary and sufficient for acquiring new spacers (19). Overexpression of these two proteins results in significant spacer acquisition into the CRISPR arrays. These findings have been demonstrated biochemically by adaptation experiments comprising Cas1, Cas2, a CRISPR array and a donor spacer (20). Furthermore, biochemical experiments showed the reverse disintegration reaction, which requires only Cas1 and a DNA substrate and thus serves as a simple tool to study the function of this protein (21).

Spacer acquisition can be a lethal process if spacers from the bacterial DNA are acquired. In *E. coli*, the negative regulator H-NS has been shown to regulate adaptation (22). H-NS represses the promoter of the operon encoding most of the Cas genes, including *cas1* and *cas2*, thus repressing adaptation. The possible catastrophic consequences of adaptation may have led to its low activity. In *E. coli* (and other bacteria), adaptation is not an efficient reaction, even when the adaptation proteins are artificially overexpressed. In such assays, only up to 20% of bacteria show spacer integration under optimal conditions after 16 h of growth (19,23). This rate is quite low for enzymatic activity. We hypothesized that inherently low activity might benefit such a hazardous protein. We therefore speculated that we could identify mutants with enhanced adaptation activity. Such mutants may also show lower fidelity in selecting spacers, as enhanced activity is often associated with lower fidelity (24).

We report here on a novel technology to identify mutants of Cas1 with enhanced adaptation features. We describe the process of their identification, validation of their *in vivo* activity, characterization of the activity of the purified proteins *in vitro* and their reduced PAM specificity.

## MATERIALS AND METHODS

### Reagents, strains and plasmids

Luria–Bertani (LB) medium (10 g/l tryptone, 5 g/l yeast extract, 5 g/l NaCl) and agar were from Acumedia. 2xYT medium contained 1.6% (w/v) bacto-tryptone (Acumedia), 1% (w/v) bacto-yeast extract (Acumedia) and 0.5% (w/v) NaCl (Acumedia) in distilled water. Antibiotics, lysozyme, l-arabinose and maltose were from Calbiochem. Restriction enzymes, ligation enzymes, DNA modification enzymes and Phusion^®^ High-Fidelity DNA Polymerase were from New England Biolabs. The bacterial strains, plasmids and oligonucleotides used in this study are listed in Supplementary Table S1.

### Plasmid construction

Plasmids were constructed using standard molecular biology techniques. DNA segments were amplified by polymerase chain reaction (PCR). Standard DNA digestions and ligations were carried out according to the manufacturer’s instructions. The primers and DNA templates used for plasmid construction are listed in Supplementary Table S2.

### Construction of bacterial strains

BW25113Δ*trxA*::kan encoding T7 RNA polymerase (T7-RNAP) under an l-arabinose-induced promoter (IYB6138) was constructed as follows: *E. coli* IYB510 (19) encoding T7-RNAP was used as the acceptor strain in P1 transduction and BW25113Δ*trxA*::kan from the Keio collection (25) served as the donor strain, as described previously (26). The kanamycin resistance marker was then excised using pCP20 (27) resulting in strain IYB6138.

### Preparation of bacteriophage T7 strains

T7 phage IYPh38, encoding *trxA-FLP* instead of gene 1 (encoding T7-RNAP), was constructed by homologous recombination as previously described (28). Briefly, wild-type T7 phage was used to infect IYB5101 cells harboring pGEM-*trxA*, a plasmid encoding *trxA-FLP* flanked by sequences homologous to those upstream and downstream of T7 gene 1. The lysate, harboring some recombinant phages that replaced gene 1 with *trxA*, was streaked on *E. coli* IYB5101 encoding gene 1 but lacking *trxA* to select the recombinant phage IYPh38. Plaques were then streak-purified on IYB5101.

### Enrichment of Cas1 and Cas2 with enhanced activity

1. *Mutant library preparation*: An overnight culture of *E. coli* IYB6138 (*trxA*^−^;T7-RNAP^+^) harboring pIY168 (Cas1–2) and a mutator plasmid MP-QUR (29) was diluted to OD_600_ ∼ 0.01 in LB supplemented with 50 μg/ml streptomycin, 50 μg/ml kanamycin and 0.2% (w/v) l-arabinose, and aerated at 37°C for 16 h. The culture was then diluted 1:10 in LB and grown at 37°C for 1 h to reach the logarithmic growth phase.
2. *Packaging Cas1–* *2 plasmids 1*: A 1 μl aliquot of T7 IYPh38 (*trxA*^+^;T7-RNAP^−^) at a multiplicity of infection (MOI) of ∼1 was then added to 0.5 ml of the culture from the previous step to pack the Cas1–2 plasmids. The infected culture was aerated for up to 2 h at 37°C until lysis occurred.
3. *Transduction and adaptation 1*: The lysate containing the packaged Cas1–2 mutant library was diluted 1:10, and 0.3 ml was added to 0.3 ml exponentially growing *E. coli* BW25113 (*trxA*^+^;T7-RNAP^−^) harboring pIY164 (spacer insertion into the array of these plasmids confers chloramphenicol resistance). The transduced culture was aerated for 1 h at 37°C to reach the logarithmic growth phase; 5 ml LB supplemented with 50 μg/ml streptomycin, 50 μg/ml kanamycin and 1 mM isopropyl β-d-1-thiogalactopyranoside (IPTG) was then added, and the culture was aerated at 32°C for 16 h to allow CRISPR adaptation.
4. *Selection and enrichment 1*: A 0.1 ml sample of the overnight culture from the previous step was spread onto LB plates supplemented with 50 μg/ml kanamycin and 34 μg/ml chloramphenicol to select for colonies that underwent spacer acquisition in the pIY164 plasmid.
5. *Packaging of Cas1–2 plasmids 2*: LB (3 ml) was spread on the plate to collect the resistant colonies. The collected colonies were diluted to OD_600_ ∼ 0.1 in 0.5 ml LB supplemented with 50 μg/ml kanamycin and were aerated at 37°C for 1 h to reach the logarithmic growth phase; 1 μl of wild-type T7 phage (*trxA*^−^;T7-RNAP^+^) at an MOI of ∼1 was then added to 0.5 ml of the culture from the previous step to pack the Cas1–2 plasmids. The infected culture was aerated for up to 2 h at 37°C until lysis occurred.
6. *Transduction and adaptation 2*: The lysate containing the packaged Cas1–2 mutant library was diluted 1:10, and 0.3 ml was added to 0.3 ml of exponentially growing *E. coli* IYB6138 (*trxA*^−^;T7-RNAP^+^) harboring pIY164. The transduced culture was aerated for 1 h at 37°C to reach the logarithmic growth phase; 5 ml LB supplemented with 50 μg/ml streptomycin, 50 μg/ml kanamycin and 1 mM IPTG was then added, and the culture was aerated at 32°C for 16 h.
7. *Selection and enrichment 2*: A 0.1 ml sample of the overnight culture from the previous step was spread onto LB plates supplemented with 50 μg/ml kanamycin and 34 μg/ml chloramphenicol to select for colonies that underwent adaptation in the pIY164 plasmid; 3 ml LB was spread on the plate to collect the resistant colonies.
8. *Repetition of the steps*: Steps 2–7 constitute a complete enrichment cycle. Altogether, we carried out eight cycles. In the first two cycles, to increase the variability of Cas1–2, we added a mutagenesis step in which the lysate prepared in step 5 was transduced into IYB6138/MP-QUR and then we proceeded to step 2. In the remaining six cycles, we followed the above protocol from step 2 to step 7 repeatedly.

### Adaptation assay using chloramphenicol resistance

Overnight cultures of *E. coli* BW25113/pIY164 harboring Cas1–2 in different variations—pIY68, pIY171, pIY183, pIY184 or pIY185—were diluted to OD_600_ ∼ 0.01 in LB supplemented with 50 μg/ml streptomycin, 50 μg/ml kanamycin and 1 mM IPTG, and were aerated at 37°C for 16 h. Cultures were then serially diluted 1:10 to 1:10^7^, and 10 μl of each dilution was plated on LB agar plates supplemented with either 50 μg/ml kanamycin or 50 μg/ml kanamycin and 34 μg/ml chloramphenicol. The fraction of chloramphenicol-resistant colonies was calculated by dividing the number of chloramphenicol-resistant colonies (grown on plates supplemented with kanamycin and chloramphenicol) by the total number of colonies (grown on plates supplemented with kanamycin only).

### Adaptation assay using PCR

Overnight cultures of *E. coli* NEB5α harboring Cas1–2 in different variations—pIY68, pIY171, pIY183, pIY184 or pIY185—were diluted to OD_600_ ∼ 0.01 in LB supplemented with 50 μg/ml kanamycin and 1 mM IPTG, and were aerated at 37°C for 16 h. A 1 μl sample from each culture then served as a template for PCR amplification using IY375Fa and IY13R. Adaptation bands were digitally captured and quantified using ImageJ software.

### Determining toxicity of Cas1-S1,S2

Overnight cultures of *E. coli* NEB5α harboring plasmids encoding the different Cas1–2 mutants were diluted to OD_600_ of 0.01 in LB supplemented with 50 μg/ml kanamycin and 1 mM IPTG. Two hundred microliters of each culture was transferred into a 96-well plate in triplicates. Cells were grown with continuous shaking at 205 rpm in a Tecan Infinite M Plex plate reader at 37°C and reads were acquired every 5 min for 14 h.

### Adaptation assay and DNA preparation for NGS

Adaptation assay for next-generation DNA sequencing (NGS) was carried out as described previously (23). Briefly, the first PCR was carried out using primers IY375Fa and IY13R. The adapted products were gel purified and used as templates for a second PCR to barcode the samples specifically and enable NGS readings, with reverse primer IY962 and forward primers IY958, IY959, IY960 or IY961. The prepared Illumina sequencing libraries were sequenced using HiSeq machines according to the manufacturer’s instructions. Samples were multiplexed in the same sequencing run. Demultiplexing was based on a 6-bp barcode that was part of the original PCR primer.

### Protein purification

Plasmids pMBP-cas1, pMBP-dcas1, pMBP-cas1-S1, pMBP-cas1-S2, pMBP-cas1-S1,S2 and pETDuet-IHF α/β were separately transformed into BL21-DE3 cells and grown in 1 l of 2xYT supplemented with 50 μg/ml kanamycin at 37°C to OD_600_ ∼ 0.5, and then induced with 0.5 mM IPTG at 18°C for 18 h. The cells were then harvested and resuspended in lysis buffer [20 mM HEPES–NaOH, pH 7.5, 500 mM KCl, 10 mM imidazole, 1 mM dithiothreitol (DTT), 0.1% Triton X-100, 1 mM phenylmethylsulfonyl fluoride (PMSF), 10% glycerol]. Sonication was used to lyse the cells and supernatant was collected after centrifugation. The cleared supernatant was subjected to a 1 ml His-Trap column (GE Healthcare) and washed with 25 column volumes of wash buffer (20 mM HEPES–NaOH, pH 7.5, 500 mM KCl, 10 mM imidazole, 1 mM DTT, 1 mM PMSF, 5% glycerol). The bound protein was then eluted with elution buffer (20 mM HEPES–NaOH, pH 7.5, 500 mM KCl, 300 mM imidazole, 1 mM DTT, 5% glycerol) and dialyzed overnight at 4°C against dialysis buffer (20 mM HEPES–NaOH, pH 7.5, 500 mM KCl, 10 mM imidazole, 1 mM DTT, 5% glycerol) in the presence of TEV protease to remove the His-maltose binding protein tag. The cleaved protein was again passed through a 1 ml His-Trap column, and flowthrough containing the cleaved protein was collected. The protein was concentrated using Amicon^®^ Ultra-4 Centrifugal Filter Units (3 kDa columns) and further purified in a Superdex 200 Increase 10/300 size-exclusion chromatography column (GE Healthcare) equilibrated with protein buffer (20 mM HEPES–NaOH, pH 7.5, 150 mM KCl, 1 mM DTT, 5% glycerol). The fractions containing the protein were pooled and concentrated using Amicon^®^ Ultra-4 Centrifugal Filter Units (3 kDa columns), snap frozen and stored at −80°C.

For purification of the Cas1–Cas2 complex, plasmids pETDuet-cas1-cas2-His, pETDuet-dcas1-cas2-His, pETDuet-cas1-S1-cas2-His, pETDuet-cas1-S2-cas2-His and pETDuet-cas1-S1,S2-cas2-His were separately transformed into BL21-DE3 cells and grown in 500 ml of 2xYT supplemented with 100 μg/ml ampicillin at 37°C to OD_600_ ∼ 0.7, and then induced with 0.5 mM IPTG at 25°C for 22 h. The cas1–cas2 integration complex was then purified as described (30).

### Integration assay

The Cy5.5-labeled 33-bp double-stranded protospacer DNA was prepared by annealing the following two strands: strand 1, CY5.5-GAAGAAGGAGATATACCATGGGGAGCGGTACAA; strand 2, CCGCTCCCCATGGTATATCTCCTTCTTCTGAGCTTGA. The DNA substrate containing the CRISPR array of *E. coli* strain B was amplified from pIY108 plasmid using OM007R and OA1R. The control DNA without the CRISPR array was amplified from pIYIT5 plasmid using IY559R and IY776. The integration assay was initiated by incubating 200 nM of the different Cas1–Cas2 complex (Cas1^WT^, dCas1, Cas1-S1, Cas1-S2 and Cas1-S1,S2) with 30 nM of protospacer at 37°C for 10 min. Following incubation, 200 nM of the IHF and 100 nM of linear DNA substrate were also added to the reaction mixture in a reaction buffer containing 50 mM KCl, 10 mM MgCl_2_, 20 mM HEPES–KOH (pH 7.5) and 1 mM DTT, and incubated at 37°C for 30 min. The reaction was stopped by adding an equal volume of loading dye containing 95% (v/v) formamide, 20 mM EDTA and 0.025% bromophenol blue, and boiled at 95°C for 10 min. The samples were then loaded onto a 10% polyacrylamide gel containing 7.5 M urea. Electrophoresis was carried out at 175 V for 1 h in 0.5× TBE buffer. Cy5.5 fluorescence was detected using a Sapphire biomolecular imager (Azure Biosystems).

### Electrophoretic mobility shift assay

Binding of the Cas1–Cas2 complex with protospacers to a plasmid encoding a CRISPR leader–repeat was studied using electrophoretic mobility shift assay (EMSA). In this assay, 400 nM of pre-annealed Cy5.5-labeled protospacers (strand 1: 5′-Cy5.5-GAAGAAGGAGATATACCATGGGGAGCGGTACAA-3′; strand 2: 5′-AACATCCGCTCCCCATGGTATATCTCCTAGAAG-3′) were first incubated with 40 nM of Cas1–Cas2 complex for 10 min at 22°C in reaction buffer containing 20 mM HEPES–NaOH (pH 7.5), 50 mM KCl, 1 mM DTT, 5 mM EDTA, 0.15% (v/v) Tween 20 and 100 g/ml bovine serum albumin. Following incubation, 7.5 nM of leader–repeat-containing plasmid (pIYIT5) was added and incubated at 22°C for an additional hour. The samples were then mixed with bromophenol blue-containing loading dye and separated in a 0.6% agarose gel in 1× TAE buffer. The gel was scanned using a Sapphire biomolecular imager (Azure Biosystems) to detect the Cy5.5 fluorescence. The agarose gel was also stained with EtBr to visualize the separated plasmid DNA bands.

### Disintegration assay

Y-DNA was prepared by annealing four single-stranded DNAs in a stepwise manner: 95°C for 5 min, 80°C for 10 min, 70°C for 10 min, 65°C for 20 min, 60°C for 10 min, 50°C for 20 min and then slowly cooling the reaction mixture at a rate of 1°C/min to room temperature. The following strands were used for Y-DNA preparation:

- Strand 1: GGCCCCAGTGCTGCAATGGA
- Strand 2: GTGAGCGTGGGTCTCCATTGCAGCACTGGGGCC
- Strand 3: Cy5.5**-**GCCCAATTTACTACTCGTTCTGGTGTTTCTCGTACCGCGAGACCCACGCT CAC
- Strand 4: TCGCGGTACGAGAAACACCAGAACGAGTAGTAAATTGGGC

The disintegration reaction was carried out in 10 μl reaction mixture containing 210 nM purified Cas1 proteins (Cas1^WT^, Cas1-S1, Cas1-S2 and Cas1-S1,S2) incubated with 40 nM Y-DNA in a reaction buffer containing 50 mM KCl, 10 mM MnCl_2_, 20 mM HEPES–KOH (pH 7.5) and 1 mM DTT. Reaction mixtures were incubated for 1 h at 37°C. Equal volumes of formamide loading dye (95% formamide, 20 mM EDTA, 0.025% bromophenol blue) were mixed with the reaction mixtures and loaded onto 10% polyacrylamide gels containing 7.5 M urea. Electrophoresis was carried out at 175 V for 1 h in 0.5× TBE buffer. Cy5.5 fluorescence was detected using a Sapphire biomolecular imager (Azure Biosystems).

## RESULTS

### Establishment of PeDPaT and enrichment of adaptation proteins with enhanced activity

**Figure 1.**
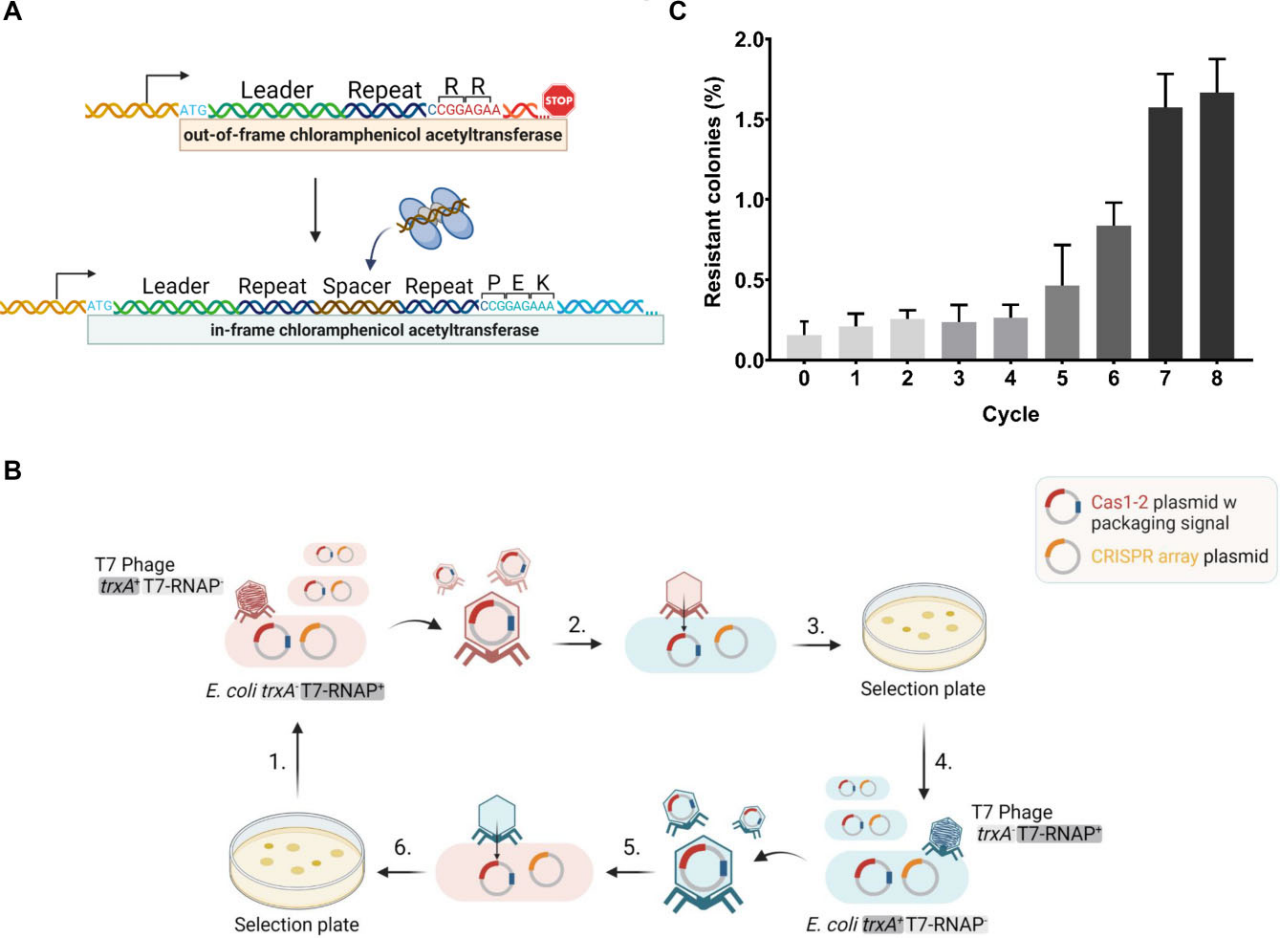
Scheme for enriching highly active Cas1 and Cas2 mutants. (**A**) DNA segment of the pIY164 plasmid conferring resistance upon spacer acquisition. An out-of-frame chloramphenicol acetyltransferase is encoded downstream of a CRISPR array. Its initiating ATG (light blue) is adjacent to the leader. Upon spacer integration, the frame is shifted due to insertion of 61-bp spacer + repeat. Resistance is consequently conferred to bacteria harboring an adapted plasmid. (**B**) PeDPaT steps detailed in the experimental procedures are depicted. (1) *Packing Cas1–2 plasmids 1*: *E. coli* IYB6138 (*trxA*^−^;T7-RNAP^+^) harboring the CRISPR-array plasmid pIY164 and a library of Cas1–2 plasmids is infected with T7 phage IYPh38 (*trxA*^+^;T7-RNAP^−^) for Cas1–2 plasmid packaging. (2) *Transduction and adaptation 1*: phage particles transduce *E. coli* BW25113 (*trxA*^+^;T7-RNAP^−^). Adaptation into pIY164 plasmid confers chloramphenicol resistance. (3) *Selection and enrichment 1*: chloramphenicol-resistant colonies are selected. (4) *Packaging of Cas1–2 plasmids 2*: colonies infected with wild-type T7 phage (*trxA*^−^;T7-RNAP^+^) are collected. (5) *Transduction and adaptation 2*: phage particles transduce *E. coli* IYB6138 (*trxA*^−^;T7-RNAP^+^). Adaptation into pIY164 plasmid confers chloramphenicol resistance. (6) *Selection and enrichment 2*: chloramphenicol-resistant colonies are selected, collected and treated as in step 1 for another cycle. (**C**) Following step 6 in each cycle, the percentage of chloramphenicol-resistant colonies out of the total colonies picked was counted and plotted as bars. Three samples were used for each point. Bars represent average ± standard deviation.

To identify Cas1 and Cas2 (hereafter Cas1–2) proteins with enhanced adaptation activity, we devised a system, which we term perpetual DNA packaging and transduction (PeDPaT), that results in the enrichment of adaptation proteins displaying enhanced activity. We used bacteria harboring plasmids that contain a CRISPR array and confer antibiotic resistance upon spacer acquisition (31). The plasmid encodes an out-of-frame chloramphenicol resistance gene; the frame is restored following adaptation of a new spacer–repeat of 61 bp (Figure 1A). We introduced plasmids encoding a library of Cas1–2 mutants into these bacteria and selected colonies that became antibiotic resistant due to adaptation. Colonies harboring Cas1–2 mutants that acquire spacers more efficiently than colonies carrying the wild-type Cas1–2 were thus enriched. Eventually, the desired Cas1–2 mutants dominated, allowing us to identify mutants with enhanced activity.

The above procedure would be easy to achieve using a sophisticated approach that allows robust DNA transductions with repeated cycles. We established PeDPaT, a system to introduce plasmids encoding the mutant proteins at high efficiency (two orders of magnitude higher than standard DNA transformation; Supplementary Figure S1), providing the ability to repeat the process with maximal plasmid transfers and minimal labor between cycles. A major obstacle that we needed to overcome is that during the packaging of plasmids encoding the T7 packaging signals by phages, the genome of the phage is also packaged, and its progeny kill the transduced bacteria. Therefore, we used a restrictive host strain that prevents the formation of the phage progeny. However, we needed phages that propagate in the bacterial strain harboring these plasmids to produce transducing phages that pack plasmids. Therefore, we also used another phage that overcame the host restriction. We alternated with restrictive and permissive strains to enable perpetual transduction cycles. Specifically, we used a phage–bacterium pair that lacked a particular gene for phage propagation (*trxA*), and another pair that lacked a different gene required for phage propagation (T7-RNAP). Each phage could propagate and package plasmids on the other bacteria. We alternated the bacteria so that packaging (which requires phage propagation) was done on the permissive strain while transduction was done on the restrictive strain (Figure 1B). We continually alternated the cycles until the desired enrichment of Cas1–2 with enhanced activity was achieved.

In the first step, plasmids encoding a library of Cas1–2 were prepared by growing the plasmids in the presence of the mutator plasmid MP-QUR (29) in *E. coli* lacking *trxA*. The plasmids were packaged by T7 phages encoding *trxA* (allowing packaging). The plasmids were then transduced into an *E. coli* strain lacking T7-RNAP (preventing lysis). As indicated above, the transduced strain encoded a CRISPR array, providing chloramphenicol resistance only upon spacer acquisition. Thus, bacteria with active Cas1–2 that acquired spacers were selected, and those with enhanced Cas1–2 adaptation were enriched. It is important to note that only the plasmids encoding Cas1–2 carry a T7 packaging signal, and thus only they are enriched in this process. In the second transduction step, T7 phages encoding T7-RNAP were used to package the enriched Cas1–2 plasmids (allowing packaging). These phage particles were then used to transduce the Cas1–2 plasmids into *E. coli* lacking *trxA* (preventing lysis). Here, the transduced strain also contained a CRISPR array that provided resistance upon adaptation. Thus, in this cycle, Cas1–2 proteins with enhanced adaptation efficiency were also enriched. Passaging the plasmids between the cycles in bacteria with a plasmid that induces mutagenesis (29) increased the variability of the proteins. The described alternating cycles were repeated eight times until the detected increase in adaptation efficiency seemed to plateau (Figure 1C). Plasmids encoding Cas1–2 from the enriched pool were then sampled and sequenced to identify the mutations enhancing adaptation.

### Validation of enhanced adaptation mutants

**Figure 2.**
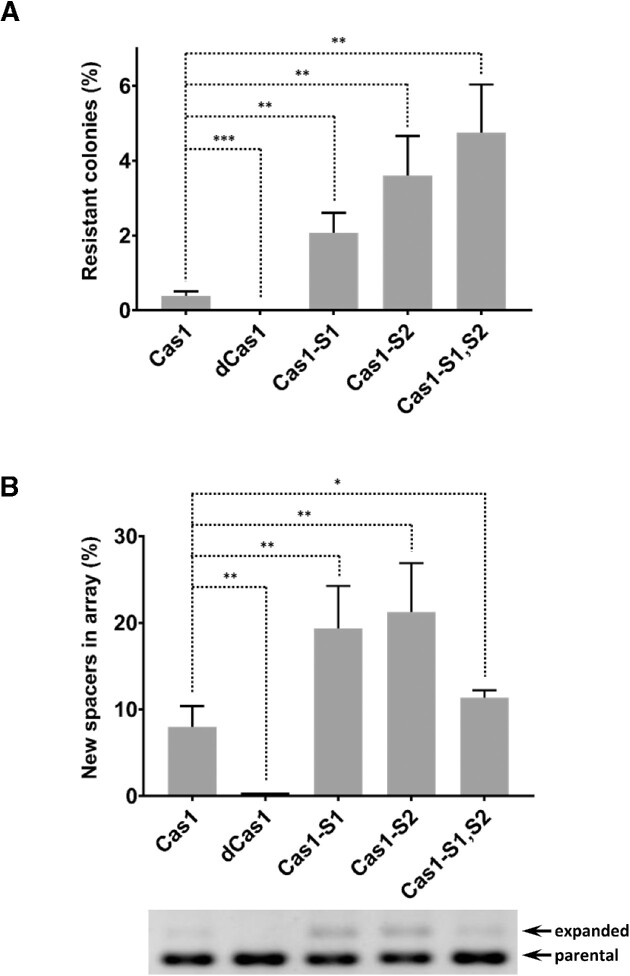
Activity of the identified Cas1 mutants. (**A**) *Escherichia coli* BW25113/pIY164 harboring the indicated Cas1 proteins were induced to allow adaptation, resulting in chloramphenicol resistance. The percentage of chloramphenicol-resistant colonies out of the total colonies is depicted. (**B**) *Escherichia coli* NEB5α harboring the indicated Cas1 proteins were induced to allow adaptation. Adaptation products were detected by PCR and gel separation. The percentage of the new spacers was calculated using ImageJ software (up). Bars represent average ± standard deviation of three independent experiments. A representative negative image of the gel is shown, corresponding to the bars in the upper panel, with the parental and expanded bands indicated by arrows (bottom). *P < 0.05; **P < 0.01; ***P < 0.001.

We isolated eight clones from the eighth cycle and sequenced their plasmids; six of them had the Cas1-E269G mutation (hereafter Cas1-S1), one had the Cas1-V76L mutation (hereafter Cas1-S2) and one had both mutations (hereafter Cas1-S1,S2). To validate that the identified mutations enhance adaptation, we introduced them into naïve plasmids and tested their adaptation efficiency using two different assays. The first determined the resistant colonies arising with each Cas1 mutant due to adaptation, compared to the wild-type Cas1. The newly constructed plasmids, encoding the Cas1 variants and Cas2, were transformed into *E. coli* harboring the CRISPR array that provides chloramphenicol resistance upon spacer acquisition. Expression of Cas1 and Cas2 was induced overnight, and the bacteria were then inoculated on LB–kanamycin–agar plates with chloramphenicol (cm^+^) or without chloramphenicol (cm^−^). The percentage of bacteria acquiring resistance (on cm^+^ plates) out of all colonies (on cm^−^ plates) was then calculated. We observed an over 4-fold increase in resistant colonies when Cas1-S1 was introduced, compared to the wild-type Cas1. Cas1-S2 showed an over 7-fold increase, and Cas1-S1,S2 showed a ∼10-fold increase in the percentage of resistant colonies (Figure 2A). As expected, the negative control, dead Cas1 (Cas1^D221A^), showed almost no resistant colonies due to lack of adaptation. We also examined the effect of substituting several residues at position 76 and determined that the V76L substitution enhances the activity more than all other tested substitutions (Supplementary Figure S2).

To further test whether the identified mutants display enhanced adaptation, we used a standard assay to measure adaptation efficiency (19). We transformed the newly constructed plasmids encoding the variants of Cas1 and Cas2 into *E. coli* bacteria encoding a CRISPR array in their genome. Following overnight Cas1–2 expression, a sample of the culture served as a template for PCR of the CRISPR array. The PCR shows a larger band corresponding to the band that acquired spacers, as well as the original non-adapted band. The ratio of the intensities of these bands is proportional to the number of colonies that underwent adaptation compared to those that did not. In this assay, Cas1-S1 and Cas1-S2 showed a significant increase of over 2- and 3-fold in adaptation compared to the wild type (Figure 2B). The dead Cas1 showed no adaptation, as expected. The double mutant Cas1-S1,S2 showed only a modest increase in adaptation compared to the wild type in this assay. We believe that some of the differences between the two assays are due to increased toxicity of the Cas1-S1,S2 plasmid (Supplementary Figure S3). The first assay is less affected by this toxicity because it positively selects the colonies that underwent adaptation, whereas the second assay is more affected because it measures the proportion of the adapted band compared to the non-adapted band; the non-adapted band also consists of colonies that lost Cas1–2 activity due to toxicity or even dead colonies. Nevertheless, both *in vivo* assays indicated that the identified mutants have enhanced adaptation activity.

### Biochemical characterization of the identified mutants

**Figure 3.**
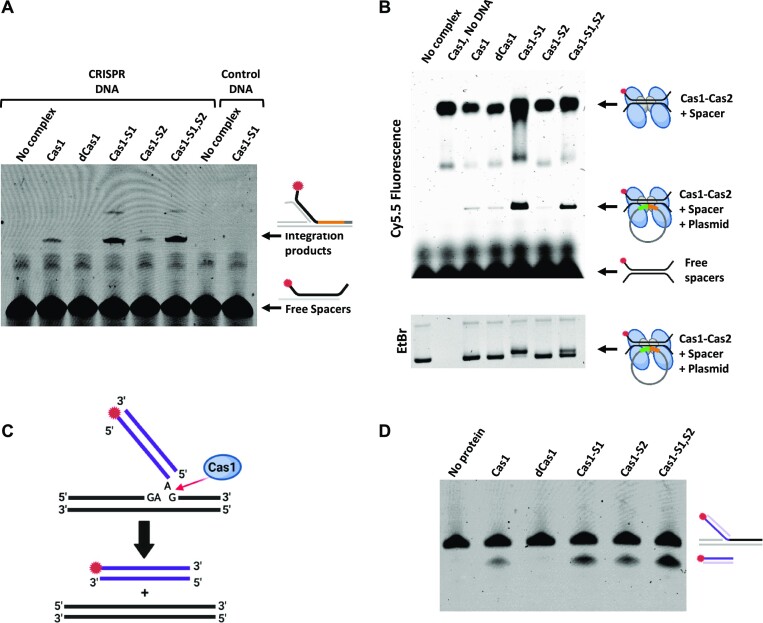
Biochemical assays to characterize the mutants. (**A**) Integration assay was carried out by incubating a complex of the indicated Cas1–Cas2 variants, IHF, Cy5.5-tagged spacer and a linear DNA encoding a CRISPR array or a control DNA in reaction buffer containing Mg^2+^. The integration products and free spacers are indicated. (**B**) EMSA was carried out by incubating a complex of the indicated Cas1–Cas2 variants, Cy5.5-tagged spacer and a plasmid encoding a CRISPR array in reaction buffer lacking Mg^2+^. The Cas1–2 complex with spacer, Cas1–2 complex with spacer and plasmid, and free spacer are indicated. Top: fluorescence imaging; bottom: ethidium bromide imaging. (**C**) Disintegration reaction is depicted. Y-DNA is disintegrated in the presence of Cas1 into two separate dsDNA products. (**D**) Disintegration assay was carried out by incubating Cas1 and a Cy5.5-tagged substrate DNA in a reaction buffer containing Mn^2+^. The original strand (53 nt) and the disintegration products (40 nt) are indicated. Quantifications and statistics of at least three independent experiments of panels (A), (B) and (D) are provided in Supplementary Figure S4.

To determine the activity of the Cas1 mutant proteins *in vitro*, we purified them in complex with Cas2, and we also purified the integration host factor (IHF) required for efficient and precise integration of spacers into the first position in a linear CRISPR array (32). We then carried out a spacer integration assay as previously described (32), with minor modifications. The Cas1–2 complexes with the different Cas1 variants were incubated with a Cy5.5-labeled DNA spacer and mixed with linear DNA containing a CRISPR array and IHF. The products were then separated by PAGE and imaged. The intensity of the integration product of Cas1-S1 and Cas1-S1,S2 was significantly higher than that of the wild type, validating the *in vivo* findings for Cas1-S1. As expected, the wild-type Cas1 showed a distinct integration band, whereas the control—dead Cas1—showed no integration (Figure 3A and Supplementary Figure S4A). Additionally, no integration bands were observed when a control DNA lacking the CRISPR was used, indicating the specificity of the integration reaction. Surprisingly, Cas1-S2 did not show enhanced activity in this assay (Figure 3A and Supplementary Figure S4A).

We next determined the capacity of the different Cas1 mutant proteins to bind DNA in an EMSA as described previously (30,33). We incubated the different Cas1–2 complexes with a Cy5.5-labeled spacer and a plasmid encoding a CRISPR array. We then separated the products by electrophoresis. The wild-type Cas1 and Cas1-S2 formed relatively weak complexes with the DNA. In contrast, Cas1-S1 and Cas1-S1,S2 showed a significantly higher complexation interaction with the plasmid DNA encoding CRISPR. These results suggest that the enhanced activity provided by the S1 mutation, also found in the Cas1-S1,S2 mutant, enables better complex formation with DNA, resulting in better adaptation (Figure 3B and Supplementary Figure S4B). It also suggests that the enhanced activity of Cas1-S2 is not related to better DNA binding.

The above *in vitro* assays examined the process of spacer integration into the CRISPR array, but not the selection and disintegration of the protospacers. To determine whether Cas1-S2 shows enhanced disintegration activity, which may explain its overall enhanced activity *in vivo*, we tested the effect of the mutations on the activity of the Cas1 mutant proteins in the initial stages, i.e. in the protospacer’s disintegration from the DNA. We utilized a disintegration assay, in which the ability of purified Cas1 alone to disintegrate a strand from the DNA is measured (21). The assay uses Y-DNA with one strand labeled with the fluorescent tag Cy5.5. Upon Cas1-mediated transesterification disintegration, the labeled strand is shortened (Figure 3C). In this assay, we observed significantly enhanced product formation for Cas1-S1, Cas1-S2 and Cas1-S1,S2 (Figure 3D and Supplementary Figure S4C), suggesting that all of these variants disintegrate the spacer better than the wild type. The enhanced activity of disintegration of Cas1-S1 may be attributed to its higher DNA binding ability, but for Cas1-S2, enhanced disintegration is seemingly the feature providing it with the enhanced adaptation activity.

### PAM conservation by the mutants compared to the wild-type Cas1

**Figure 4.**
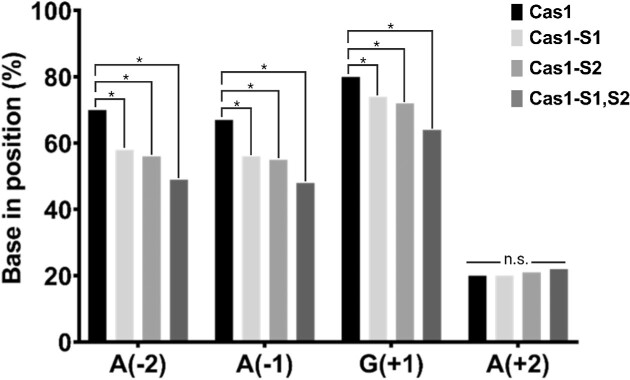
Abundance of bases at the consensus PAM and the adjacent nucleotide (positions −2 to +2). Each bar represents the percentage of the indicated base of the spacer (the left three bases represent the PAM consensus motif at positions −2 to +1; the rightmost base is an A base located at position +2 of the spacer) acquired by the different Cas1 mutants. The number of unique spacer reads used for the analysis for each protein is 256 751 for wt-Cas1, 369 828 for Cas1-S1, 352 051 for Cas1-S2 and 251 462 for Cas1-S1,S2. *P < 10^−5^; n.s., nonsignificant.

Lastly, we sequenced the spacers integrated by the different Cas1 proteins. We performed naïve adaptation assays (19) by inducing the different adaptation proteins under similar conditions. We then extracted the DNA and performed a PCR on the CRISPR array. The visible band with the highest molecular weight, encoding the new adaptation products, was extracted and prepared for NGS. We analyzed the sequencing results by comparing the relative abundance of each nucleotide in the newly adapted spacers at every position, starting from the −2 position (PAM) to the +33 position (end of the spacer). We observed that while the different Cas1 variants showed no significant differences in the abundance of nucleotides at positions +2 to +33, the abundance of nucleotides at positions −2, −1 and +1, constituting the PAM, changed significantly. In the −2, −1 and +1 positions, all three variants displayed significantly lower preference for the respective nucleotide comprising the PAM compared to the wild-type Cas1 (Figure 4 and Supplementary Figure S5). These results indicate that the mutations decrease the fidelity of the proteins in selecting spacers with a correct PAM.

## DISCUSSION

In this work, we developed a highly useful technology that perpetually transduces plasmids robustly and with minimal labor. The technology takes advantage of the unique growth requirement of T7 bacteriophage and its robust transduction ability to allow alternating cycles of efficient plasmid transfer without killing the bacteria. We used these robust transductions of plasmids, with repeated cycles, which we termed PeDPaT, to enrich and search for proteins with high adaptation activity, and we further characterized them biochemically. The technology allows for large-scale DNA transfer, as opposed to electroporation where only small volumes can be transferred. In addition, plasmid loss between transfers is minimal due to the efficiency of phage transduction. This robustness and effectiveness enable the enrichment of desired features with a minimum number of cycles. Furthermore, there is minimal effort between alternating cycles, because the technology does not require plasmid extraction or the preparation of electrocompetent bacteria, but merely phage and bacterial propagation. We believe that the technology of this efficient and perpetual plasmid delivery system could be extended to many other screens requiring these features.

Other approaches using phage transductions and phagemids also enable efficient DNA transfer into bacteria. A notable one is the Phage-Assisted Continuous Evolution that links a protein function to the ability of phage to propagate, thus allowing continuous evolution of a particular protein feature (29). However, this approach does not enable repetitive packaging and retransduction of the DNA into another bacteria, a property required for selecting and enriching certain features that cannot be directly linked to a phage’s ability to propagate.

We have used the PeDPaT technology to enrich Cas1–2 mutants displaying enhanced activity. Shipman *et al.* have used an elegant directed evolution approach to change the PAM specificity of the Cas1–2 complex (34). They cloned a mutant library of Cas1–2 next to a CRISPR array and facilitated the acquisition of spacers with PAM and non-PAM sequences by electroporating them into the bacteria. They then isolated the Cas1–2 mutants that incorporated the non-PAMs by amplifying the plasmids that encoded them only if they were adjacent to the non-PAM sequence using PCR with oligos specific to these non-PAM sequences. This approach yielded the desired mutants with decreased canonical PAM specificity. Using this approach should theoretically yield Cas1–2 proteins with enhanced activity if repeated for many cycles because amplifying a library of Cas1–2 mutants only if a spacer is inserted would enrich Cas1–2 with enhanced activity. However, changing the PAM can be facilitated by electroporation of specific spacers, whereas enriching for enhanced activity cannot be facilitated similarly. The electroporation of spacers facilitated the selection of desired mutants following only a few cycles. In our case, presumably more cycles were required (as there is no procedure that facilitates the evolution), encompassing PCR, cloning and transformation of the ligated plasmids, and therefore we preferred the workflow of the PeDPaT technology that required merely transductions.

**Figure 5.**
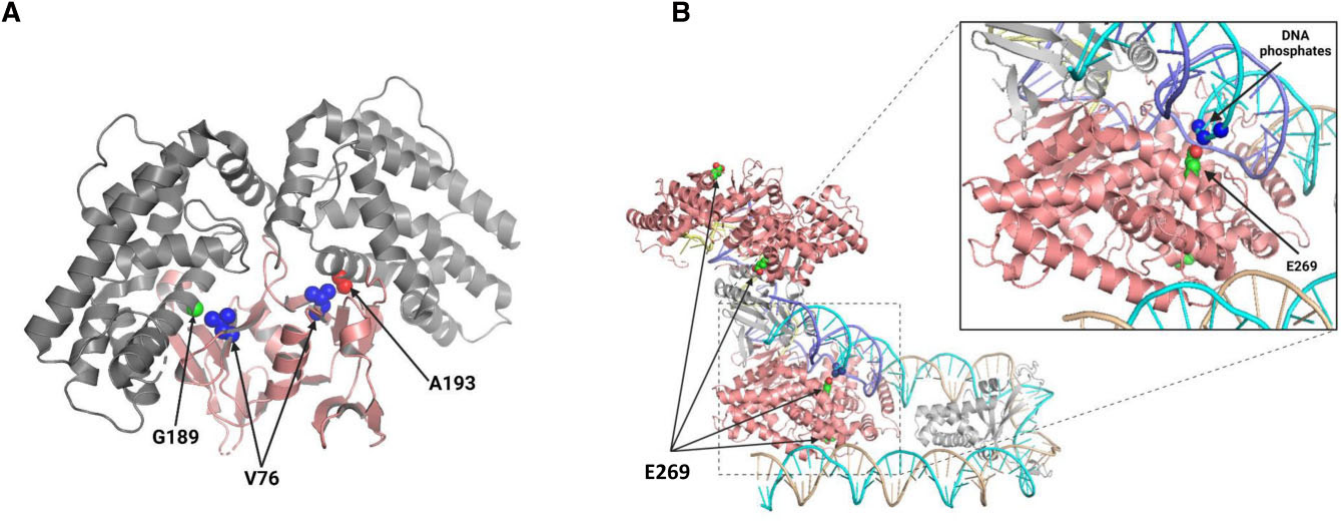
Structural context of the mutations that enhance Cas1 activity. (**A**) Face view of the *E. coli* Cas1 dimer structure (pdb 3NKD): C-terminal domain (alpha) in gray; N-terminal domain (beta) in pink. Arrows point to the relevant residues discussed in the text. (**B**) Face view of the Cas1–Cas2–IHF–DNA holo-complex (pdb 5WFE): Cas1 in pink; DNA strands in ochre, cyan, purple and yellow; Cas2 and IHF in light gray. Arrows point to the relevant residues discussed in the text.

The mutations that we identified as enhancing Cas1 activity are at positions 269 and 76. A possible rationalization for the effects of the E269G substitution (Cas-S1) might be gleaned from the structure of the holo-complex of Cas1–Cas2 with a spacer and integration-site DNA (pdb 5WFE) (17). We find that in one of the Cas1 monomers, E269 protrudes toward the integration-site DNA (Figure 5A). Therefore, substitution to glycine eliminates the repulsion between a phosphate group and the negatively charged glutamate, stabilizing the binding to DNA. Indeed, the EMSA showed increased DNA binding by this variant (Figure 3B). However, additional effects of this substitution cannot be excluded; notably, changing E269 to glycine might also destabilize the helix that harbors this residue. Interestingly, in Cas1 enzymes from other organisms (e.g. *Streptococcus pyogenes*, *Pseudomonas aeruginosa* and *Archaeoglobus fulgidus*), a positive residue often appears in the homologous position (35), further stabilizing the contact with integration-site DNA. We believe that both effects, DNA binding and helix destabilization, contribute to improved integration efficiency.

The site of the substitution V76L lies at the interface between the two Cas1 domains—alpha and beta (Figure 5B). The architecture of each monomer of Cas1 consists of a beta-sandwich-like N-terminal domain (beta) connected via a short linker to a larger, primarily alpha-helical, C-terminal domain (alpha) that harbors the catalytic residues. Notably, the short linker is a hinge that allows the two domains to be positioned in different relative orientations in the two monomers. The V76 residue is on the beta domain opposite residues G189 and A193 in the two monomers, which are part of a helix from the alpha domain. The substitution to a larger hydrophobic residue could form van der Waals forces with the opposite alanine at position 193 in one monomer to stabilize the contact between the two domains. These forces might affect the dynamics of Cas1 afforded by the linker between the domains. In the case of the other monomer, the amino acid opposite V76 is a glycine at position 189, which is a rare residue in alpha helices because it destabilizes this secondary structure conformation to transiently or locally assume a less compact state (36,37). Thus, V76L seems to be an example of a substitution, located far from the active site, which exerts its allosteric effect by altering the inner dynamics and conformational landscape of the Cas1 dimer.

The biochemical assays (Figure 3) showed enhanced DNA binding, and enhanced integration by the Cas-S1 variant (and the combined variant) but not by the Cas1-S2 variant. However, the Cas1-S2 variant showed enhanced disintegration activity. The enhanced disintegration activity observed for the Cas1-S1 variant may be attributed to its enhanced DNA binding, whereas the enhanced disintegration of the Cas1-S2 variant cannot be attributed to enhanced DNA binding. We therefore propose that the enhanced activity *in vivo* of both proteins stems from two distinct mechanisms—enhanced DNA binding for Cas1-S1 and enhanced disintegration activity for Cas1-S2. The enhanced DNA binding activity allows Cas1-S1 to both retrieve more spacers and integrate them more efficiently into the CRISPR array. The increased disintegration activity of Cas1-S2 allows it to retrieve spacers more efficiently than the wild type, and thus have enhanced overall integration activity.

While the specific activity of the adaptation proteins significantly increased, the specificity of PAM selection significantly decreased. The decrease in specificity suggests that a relatively low level of Cas1–2 activity is essential for maintaining specificity. In many systems, higher activity comes at the expense of higher fidelity, and vice versa. For example, a recent report showed remarkably specific cleavage activity of the Cas9 protein, accompanied by extremely low activity (37). This phenomenon of specificity versus activity is also widespread in other systems; the HIV reverse transcriptase (RT) does not discriminate well against incorporating nucleotide analogs, thus conferring the virus’s high fitness due to frequent mutations. Mutant RTs that discriminate better against analogs have higher specificity to their substrate, but reduce the fitness of the virus (24). It is possible that complex simultaneous mutations in Cas1 would enhance the activity while preserving the PAM specificity, but such mutations were not found in our screen.

The driving hypothesis of this study was that some mutations in the adaptation proteins may downregulate their activity due to their possible hazardous nature. Although we cannot decisively conclude that the identified residues indeed regulate Cas1 activity, we carried out an experiment showing that several residues at position 76 enhance its activity over that of the wild type (Supplementary Figure S2). Interestingly, in another *E. coli* (strain 2021CK-01826; BioProject ID PRJNA288601; BioSample accession SAMN24965743), there is perfect homology of all residues to K-12 Cas1, except at position 76, where the valine is replaced by isoleucine, a residue that enhances the activity. This finding further suggests that position 76 plays a major role in determining adaptation activity.

To the best of our knowledge, this is the first example of a mutation enhancing Cas1 activity in any bacterial species (https://ecocyc.org/gene?orgid=ECOLI&id=G7425-MONOMER#tab=FTRS; https://www.uniprot.org/uniprotkb?query=cas1). The identified proteins may be used for applications requiring robust adaptation, such as a molecular machinery for recording biological events, or even for storing digital images and movies in DNA (34,38–42). We believe that our reported approach, PeDPaT, may be used to identify robust adaptation proteins from other species as well, and thus enrich the molecular toolbox for future applications even further.

## Supplementary Material

gkad510_Supplemental_File

## Data Availability

The NGS data of the spacers generated in this study are available at the National Center for Biotechnology Information’s Sequence Read Archive with the following accession codes: BioProject ID PRJNA975318; BioSample accession SAMN35324103.
