# Supplementary material for: Highly active CRISPR-adaptation proteins revealed by a robust enrichment technology
Source: Nucleic Acids Res. 2023 Jun 16;51(14):7552–62. doi: 10.1093/nar/gkad510 (PMC10415146; doi:10.1093/nar/gkad510)
Supplement: gkad510_Supplemental_File [file gkad510_supplemental_file.pdf]

SUPPLEMENTARY INFORMATION

SUPPLEMENTARY FIGURES

Figure S1

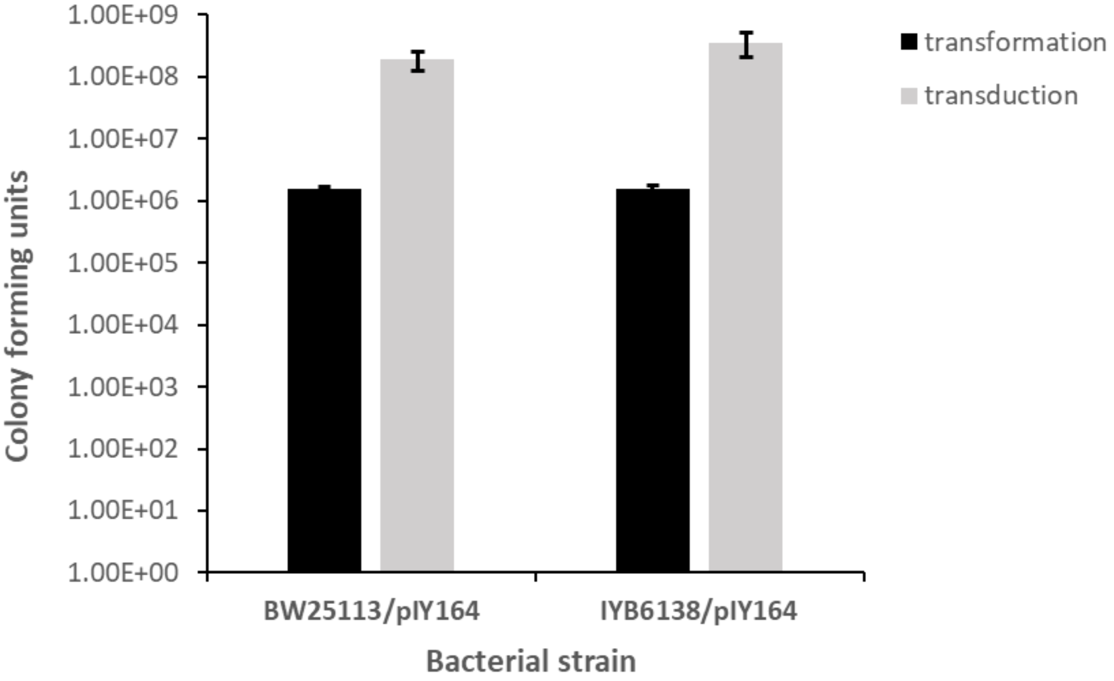

Comparing electroporation and transduction efficiencies. *E. coli* strains BW25113 or IYB6138 harboring the pIY164 plasmid were either electroporated or transduced with a pIY168 plasmid. Cultures were then diluted and the number of transformants or transductants (colony forming units) was determined. Bars represent the average ± SD of three independent experiments. Statistical significance of $p < 0.05$ was calculated for electroporation compared to transduction by unpaired, two-tailed Student's t test.

Figure S2

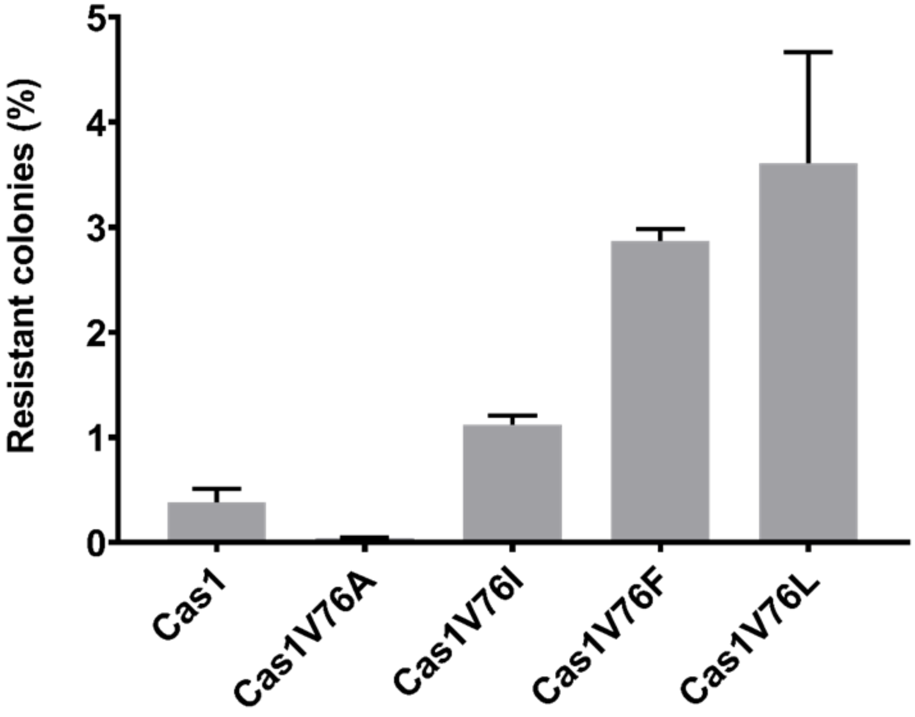

Effect of different substitutions of the Cas1 V76 residue. *E. coli* BW25113/pIY164 cells harboring the indicated Cas1 mutants were induced to allow adaptation, resulting in chloramphenicol resistance. The percentage of chloramphenicol-resistant colonies out of the total colonies is depicted. Bars represent average ± standard deviation of 3 independent experiments.

Figure S3

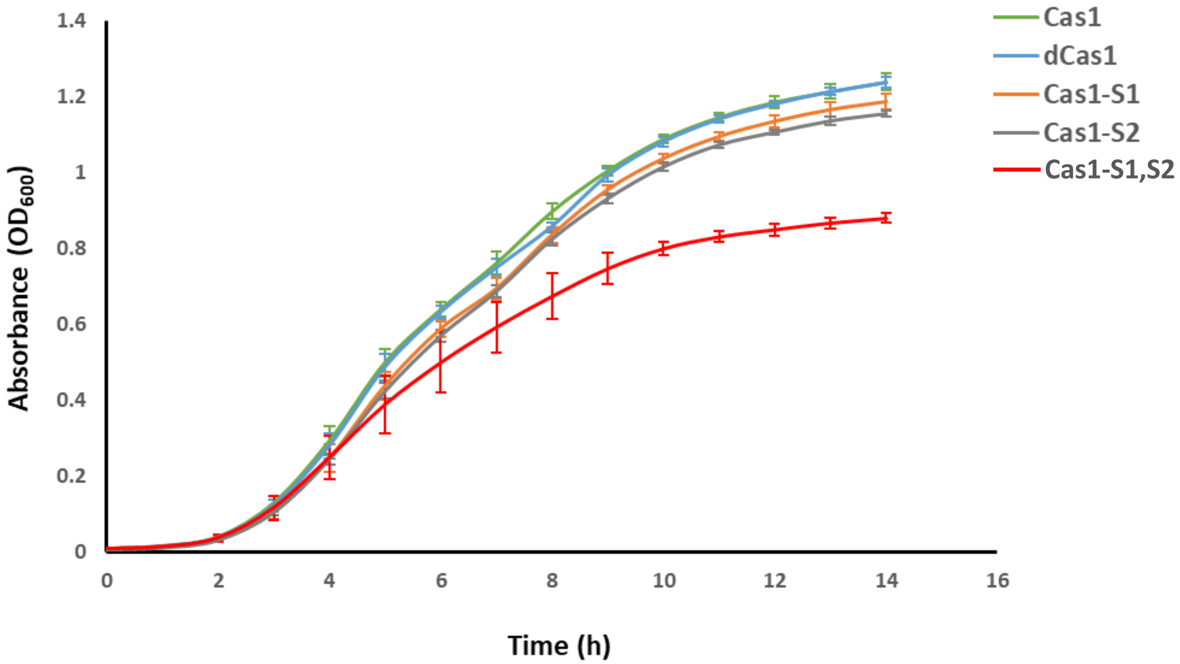

Toxicity of the Cas1-S1,S2 protein. Growth curves of *E. coli* cultures expressing the indicated Cas proteins were generated as described in the Materials and Methods section. Graphs represent the average of three independent experiments with standard deviations calculated for each 1 h point. $p = 0.0001$ for cultures harboring Cas1-S1,S2 compared to wt-Cas1 at timepoint 14 h as determined by unpaired, two-tailed Student's t test.

Figure S4

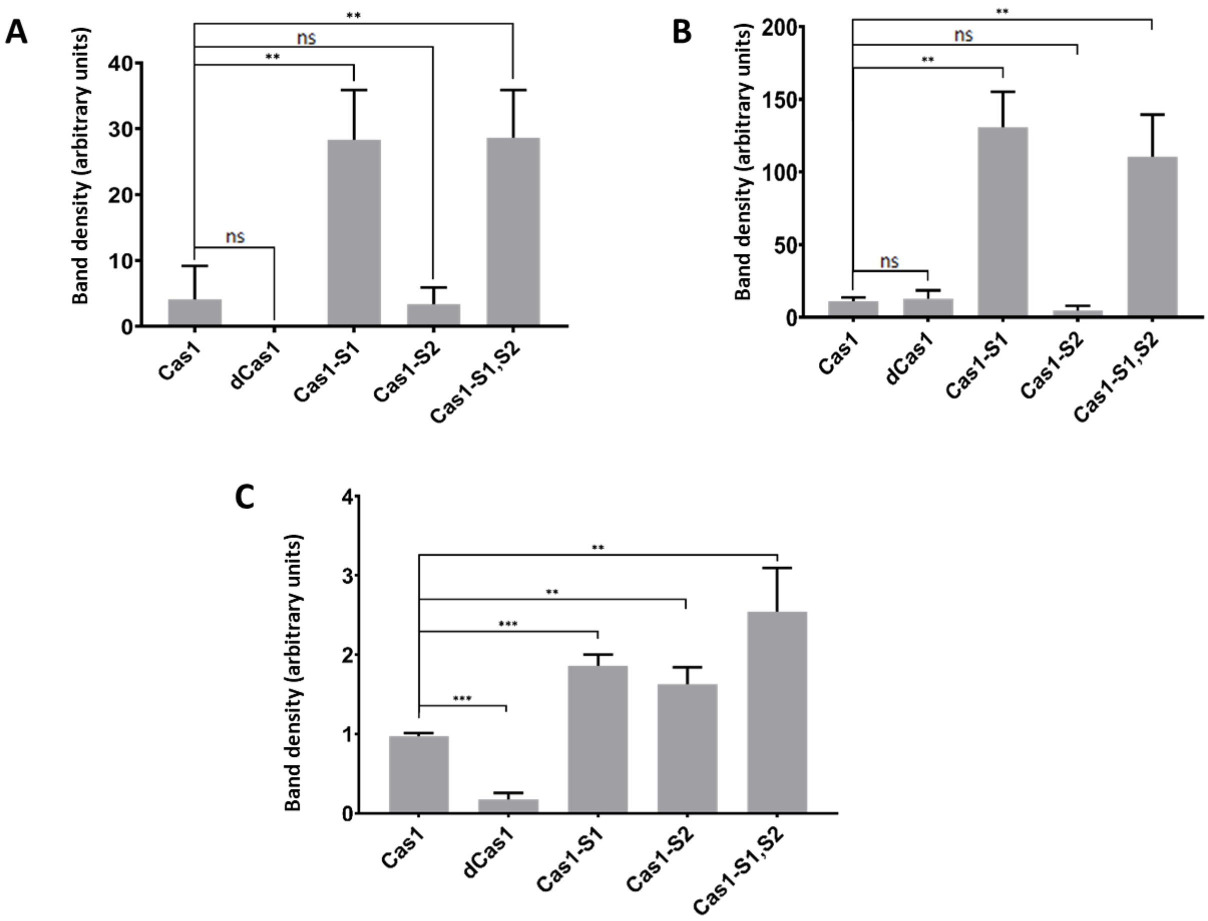

ImageJ quantification for the different assays presented in Figure 3. (A) Quantification of the integration assay whose representative image is shown in Figure 3A. (B) Quantification of the EMSA assay whose representative image is shown in Figure 3B (top, Cy5.5 fluorescence). (C) Quantification of the disintegration assay whose representative image is shown in Figure 3D.

ns, not significant; ***p* < 0.005; ****p* < 0.0005. Statistical significance was calculated for all variants compared to Cas1 by unpaired, two-tailed Student's *t* test. All assays whose statistics are shown were independently repeated at least 3 times. The control DNA (no CRISPR) in figure 3A was carried out once.

Figure S5

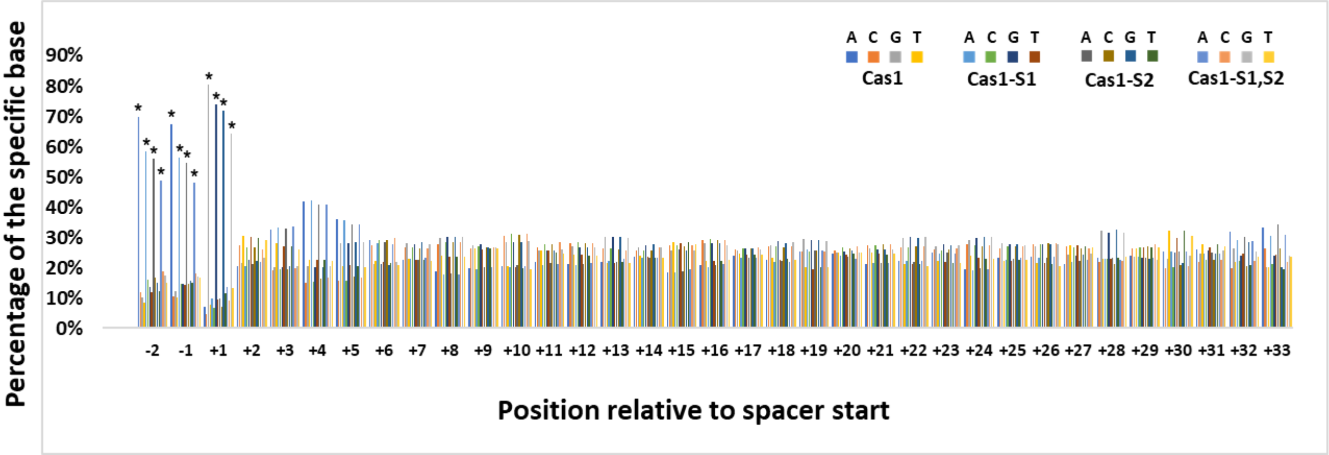

Abundance of each base in the acquired spacers. Each bar represents the percentage of a specific base at the indicated position of the spacer acquired by the different Cas1 mutants. *$p < 10^{-5}$.

SUPPLEMENTARY TABLES

Table S1. Bacterial strains, plasmids and oligonucleotides used in this study

**Bacterial/Phage strains**	**Description/sequence**	**Source or reference**
NEB5α	F⁻ ϕ80*lacZ*ΔM15Δ*(lacZYA-argF*) U169 *deoR recA1 endA1 hsdR17* (r_k^-, m_k^+) *gal*⁻ *phoA supE44* λ⁻ *thi* ⁻*1 gyrA96 relA1*	New England Biolabs
BW25113	F⁻ Δ(*araD-araB)567* Δ*lacZ4787*(::rrnB-3) λ⁻ *rph-1* Δ(*rhaD-rhaB*)*568 hsdR514*	(1)
BL21-AI	F⁻ *ompT hsdSB*(rB–, mB–) *gal dcm araB*::*T7RNAP-tetA*, tet^r	Invitrogen
IYB5101	BW25113 *araB*::T7-RNAp-tetA, tet^r	(2)
IYB6138	IYB5101 Δ*trxA*::kan^r	This study
Wild-type T7	Wild-type	Lab collection
IYPh38	T7Δ*gp1::trxA*	
**Plasmids**		
pCA24N	pUC, cm^r	(3)
pCas1+2	pCDF-1b (Novagen) cloned with *cas1,2* under T7 promoter, str^r	(4)
pWUR-IPTG-cas1+2	CloDF13 cloned with *cas1,2* under T5-lac promoter, str^r	This study
pIY040	CloDF13 cloned with *cas1,2* under tac promoter, str^r	This study
pLR053	p15A cloned with T7 packaging signal, kan^r	This study
pIY168	p15A cloned with *cas1,2* under tac promoter, T7 packaging signal, kan^r	This study
pIY171	p15A cloned with *cas1*^{D221A}*,2* under tac promoter, T7 packaging signal, kan^r	This study
pIY183	p15A cloned with *cas 1*^{E269G}*,2* under tac promoter, T7 packaging signal, kan^r	This study
pIY184	p15A cloned with *cas1*^{V76L}*,2* under tac promoter, T7 packaging signal, kan^r	This study
pIY185	p15A cloned with *cas1*$^{V76L/E269G}$*,2* under tac promoter, T7 packaging signal, kan^r	This study
pIYGS2	pUC cloned with mutated leader-repeat and out of frame cm^r, kan^r	This study
pIY128	CloDF13 cloned with mutated leader-repeat and out of frame cm^r, st^r	This study
pIY164	CloDF13 cloned with J23119 promoter, mutated leader-repeat and out of frame cm^r, st^r	This study
pT7-5-NG+1	pUC cloned with ftsY NG domain, amp^r	(5)
pACYC177	P15A cloned with T7 packaging signal, amp^r, kan^r	Lab collection
MP-QUR	CloDF13 cloned with mutators genes under arabinose promoter, st^r	(6)
pET His6 MBP TEV LIC	pUC cloned with His-MBP-TEV, kan^r	addgene Plasmid #29708

pMBP-*cas1*	*cas1* cloned with His-MBP-TEV-*cas1*, kan^r	This study
pMBP-*dcas1*	$cas1^{D221A}$ cloned with His-MBP-TEV-$cas1^{V76L}$, kan^r	This study
pMBP- $cas1^{V76L}$	$cas1^{V76L}$ cloned with His-MBP-TEV-$cas1^{V76L}$, kan^r	This study
pMBP- $cas1^{E269G}$	$cas1^{E269G}$ cloned with His-MBP-TEV-$cas1^{E269G}$, kan^r	This study
pMBP- $cas1^{V76L/E269G}$	$cas1^{V76L/E269G}$ cloned with His-MBP-TEV-$cas1^{V76L/E269G}$, kan^r	This study
pIYIT5	plasmid with leader-repeat, kan^r	This study
pETDuet1- cas2-His	cas2 cloned in pETDuet1 with His tag at C-terminal, amp^r	This study
pETDuet1- *cas1*-cas2-His	cas1 cloned in pETDuet-1-cas2-His, amp^r	This study
pETDuet1-*dcas1*-cas2-His	$cas1^{D221A}$ cloned in pETDuet-1-cas2-His, amp^r	This study
pETDuet1-$cas1^{V76L}$ -cas2-His	$cas1^{V76L}$ cloned in pETDuet-1-cas2-His, amp^r	This study
pETDuet1-$cas1^{E269G}$ -cas2-His	$cas1^{E269G}$ cloned in pETDuet-1-cas2-His, amp^r	This study
pETDuet1- $cas1^{V76L/E269G}$ -cas2-His	$cas1^{V76L/E269G}$ cloned in pETDuet-1-cas2-His, amp^r	This study
pETDuet1- *IHFβ*	*IHFβ* cloned in pETDuet1, amp^r	This study
pETDuet1-*IHFα/β*	*IHFα* cloned in pETDuet1- *IHFβ* with N-terminal His tag, amp^r	This study
pIY108	plasmid with leader-repeat, kan^r	This study
**DNA for integration assay**		
CRISPR DNA	amplify pIY108 using OM007R and OA1R	This study
Control DNA	amplify pIYIT5 using IY559R and IY776	This study
**Oligonucleotides**	**5'→3'**	
IY13R	CATCACCTTTGGCTTCGGCTG	
IY143F	CAATAAACCGGTAAACCAGC	
IY212F	CTGCGCTAGTAGACGAGTCC	
IY349R	TGCGAATGACCTTGAGTTTGTCCCTCTATAGTGAGTCGTATTGATTTGGCG TGTAGGCTGGAGCTGCTTC	
IY353F	TCTGCGGGTGGCCTGAATAGGTACGATTTACTAACTGGAAGAGGCACTAA GATCCGTCAGCCTGCAGTTC	
IY375Fa	GATCAAGCTTGGAAACAAAGAATTAGCTGATC	
IY413F	TTTCCATAGGCTCCGCCCCC	
IY544R	TTACGCCCCGCCCTGCCACT	
IY554F	ACGTTGTGTCTCAAAATCTCTGATG	
IY563F	AGGTCGAGGGTGAAGTACTTGC	

IY563R	CGTTAGGAGGTGACTTTAGGAGG
IY56F	ATGACCTGGCTTCCCCTTAA
IY808	TTGACAGCTAGCTCAGTCCTAGGTATAATGCTAGCACTAGTGAAAGAGGA GAAATACTAG
IY840	CTAGTGAAAGAGGAGAAATACTAGATG
IY88F	CCATCATTAAATTTGACACTGT
IY88R	CTGCAATATCGTAAACAAAGG
IY936	GGGACGTGCTTGCCGCTGGA
IY937	CTATAAGCGGAATCAATTTGGC
IY938	CTATGGGTGGGGGAAGCGGG
IY939	CAACAATGTTCCAACTTGCG
IY958	ACACTCTTTCCCTACACGACGCTCTTCCGATCTNNNNATCACGGAACAAAT GTATACTTTTAGAGAG
IY959	ACACTCTTTCCCTACACGACGCTCTTCCGATCTNNNNCGATGTGAACAAAT GTATACTTTTAGAGAG
IY960	ACACTCTTTCCCTACACGACGCTCTTCCGATCTNNNNTTAGGCGAACAAAT GTATACTTTTAGAGAG
IY961	ACACTCTTTCCCTACACGACGCTCTTCCGATCTNNNNTGACCAGAACAAAT GTATACTTTTAGAGAG
IY962	GTGACTGGAGTTCAGACGTGTGCTCTTCCGATCTNNNNCATCACCTTTGGC TTCGGCTG
LR53F	CTAGGATCCACATGTACTATCAAGTCAGC
LR53R	TCAGGATCCACATGTACTACTTGAGATCGTT
MG274R	CGATCACTGCCCGCTTTCCAGTC
MG37F	GTCAAGTCAGCGTAATGCTC
OA11R	AGTTAATTTCTCCTCTTTAA
OA12F	ATTTCCTAATGCAGGAGTCG
OA2R	ATGGGGCTGACTTCAGGTGC
RG260F	TTGACAATTAATCATCGGCTCGTATAATGTGTGGAATTGTGAGCGGAT
RG260R	GGAATACTGTTTCCTGTGTGAAATTGTTATCCGCTCACAATTCCACAC
SM18F	CGTCTTCACCTCGAGAAATC
TM224F	GATAACGGATCCGAATTCGAGCG
TM224R	GGATTGGAAGTACAGGTTTTCCTCG
TM486F	Cy 5.5-GAAGAAGGAGATATACCATGGGGAGCGGTACAA
TM489	Cy5.5- GCCCAATTTACTACTCGTTCTGGTGTTTCTCGTACCGCGAGACCCACGCTCA C

TM494R	CCGCTCCCCATGGTATATCTCCTTCTTCTGGGC
TM498	GGCCCCAGTGCTGCAATGGA
TM524	GTGAGCGTGGGTCTCCATTGCAGCACTGGGGCC
TM525	TCGCGGTACGAGAAACACCAGAACGAGTAGTAAATTGGGC
TM485F	GAAAACCTGTACTTCCAATCCATGACCTGGCTTCCCCTTAATCC
TM485R	CTCGAATTCGGATCCGTTATCTCAGCTACTCCGATGGCCTGCATC
OM007R	AAAAGTTGGTAGATTGTGACTGG
OA1R	TAATCAATGGATTAAGTACT
TM589F	AGTATAAGAAGGAGATATACATatgagtatgttggtcgtggtcac
TM589R	ggtgatggtgagaagatttagaagaaccaacaggtaaaaaagacaccaaccttaaac
TM590F	tctaaatcttctcaccatcaccatcaccattgaCTCGAGTCTGGTAAAGAAACC
TM591F	AACTTTAAGAAGGAGATATACCATGACCTGGCTTCCCCTTAATCC
TM591R	TTCGACTTAAGCATTATGCGGCTCAGCTACTCCGATGGCCTG
TM592R	CATGGTATATCTCCTTCTTAAAGTTAAACAAAATTATTTCTAGAG
TM382R	CATATGTATATCTCCTTCTTATACTTAAC
TM558R	AACATCCGCTCCCCATGGTATATCTCCTAGAAG
TM611R	CCGCTCCCCATGGTATATCTCCTTCTTCTGAGCTTGA
IY559R	TTGTCGCACCTGATTGCCCG
IY776	AGCCATATTCAACGGGAAACGTC
TM383F	AAGAAGGAGATATACATATGaccaagtcagaattgatagaaagac
TM383R	GTTTCTTTACCAGACTCGAGttaaccgtaaatattggcgcgatcg
TM382F	CTCGAGTCTGGTAAAGAAACC
IY971	GGATCCGGAAAACCTGTATTTTCAGGGCTCCatggcgcttacaaaagctgaaatgtc
IY972	CGACTTAAGCATTATGCGGCttactcgtctttgggcgaag
IY969	GCCGCATAATGCTTAAGTCGA
IY970	AATACAGGTTTTCCGGATCCTGGCTGTGGTGATGATGGTGATGGCTGCTG<br>CCCATGG

Table S2. Plasmids construction by the indicated primers and templates

Plasmid	DNA template 1	Primers 1	DNA template 2	Primers 2	Comments
**pWUR-IPTG-cas1+2**	pCas1+2	IY56F/OA12F	pCA24N	SM18F/OA11R	ligation
**pIY040**	pWUR-IPTG-cas1+2	IY56F/IY409R	pT7-5-NG+1	RG260F/RG260R	ligation
**pLR053**	pACYC177	MG37F/IY413R	T7	IY563F/IY563R	ligation
**pIY168**	pLR053	IY413F/IY554F	pIY040	IY212F/MG274R	ligation
**pIY171**	pIY168	IY88F/IY88R			Site-directed mutagenesis
**pIY183**	pIY168	IY936/IY937			Site-directed mutagenesis
**pIY184**	pIY168	IY938/IY939			Site-directed mutagenesis
**pIY185**	pIY183	IY938/IY939			Site-directed mutagenesis
**pMBP-*cas1***	pIY168	TM224F/R	pET His6 MBP TEV LIC	TM485F/R	Gibson assembly
**pMBP-*dcas1***	pIY171				
**pMBP-*cas1*^{V76L}**	pIY184	TM224F/R	pET His6 MBP TEV LIC	TM485F/R	Gibson assembly
**pMBP-*cas1*^{E269G}**	pIY183	TM224F/R	pET His6 MBP TEV LIC	TM485F/R	Gibson assembly
**pMBP-*cas1*$^{V76L/E269G}$**	pIY185	TM224F/R	pET His6 MBP TEV LIC	TM485F/R	Gibson assembly
**pIY128**	pCas1+2	IY143F/OA2R	IYGS2	IY840/IY544R	ligation
**pIY164**	pIY128	IY143F/IY808			self-ligation
**pIYSG2**					gene synthesis (Genescript)
**pIYIT5**					gene synthesis (Genescript)
**pETDuet1-*cas2*-His**	pWUR-IPTG-cas1+2	TM589F/R	pETDuet1	TM590F/TM382R	Gibson assembly
**pETDuet1-*cas1*-*cas2*-His**	pIY168	TM591F/R	pETDuet1- *cas2*-His	IY969/TM592R	Gibson assembly
**pETDuet1-*dcas1*-*cas2*-His**	pIY171	TM591F/R	pETDuet1- *cas2*-His	IY969/TM592R	Gibson assembly
**pETDuet1-*cas1*^{V76L} -*cas2*-His**	pIY183	TM591F/R	pETDuet1- *cas2*-His	IY969/TM592R	Gibson assembly
**pETDuet1-*cas1*^{E269G} -*cas2*-His**	pIY184	TM591F/R	pETDuet1- *cas2*-His	IY969/TM592R	Gibson assembly
**pETDuet1-*cas1*$^{V76L/E269G}$ -*cas2*-His**	pIY185	TM591F/R	pETDuet1- *cas2*-His	IY969/TM592R	Gibson assembly
**pETDuet1-*IHFβ***	BW25113 genomic DNA	TM383F/R	pETDuet1	TM382F/R	Gibson assembly
**pETDuet1-*IHFα/β***	BW25113 genomic DNA	IY571/IY572	pETDuet1-*IHFβ*	IY970/IY969	Gibson assembly

References

1. Datsenko, K.A. and Wanner, B.L. (2000) One-step inactivation of chromosomal genes in Escherichia coli K-12 using PCR products. *Proc Natl Acad Sci U S A*, **97**, 6640-6645.
2. Yosef, I., Goren, M.G., Kiro, R., Edgar, R. and Qimron, U. (2011) High-temperature protein G is essential for activity of the Escherichia coli clustered regularly interspaced short palindromic repeats (CRISPR)/Cas system. *Proc Natl Acad Sci U S A*.
3. Kitagawa, M., Ara, T., Arifuzzaman, M., Ioka-Nakamichi, T., Inamoto, E., Toyonaga, H. and Mori, H. (2005) Complete set of ORF clones of Escherichia coli ASKA library (A Complete Set of E. coli K-12 ORF Archive): Unique Resources for Biological Research. *DNA Res*, **12**, 291-299.
4. Yosef, I., Goren, M.G. and Qimron, U. (2012) Proteins and DNA elements essential for the CRISPR adaptation process in Escherichia coli. *Nucleic Acids Res*, **40**, 5569-5576.
5. Bercovich-Kinori, A. and Bibi, E. (2015) Co-translational membrane association of the Escherichia coli SRP receptor. *J Cell Sci*, **128**, 1444-1452.
6. Esvelt, K.M., Carlson, J.C. and Liu, D.R. (2011) A system for the continuous directed evolution of biomolecules. *Nature*, **472**, 499-503.
